# Immunomodulatory Efficacy-Mediated Anti-HCV and Anti-HBV Potential of Kefir Grains; Unveiling the In Vitro Antibacterial, Antifungal, and Wound Healing Activities

**DOI:** 10.3390/molecules27062016

**Published:** 2022-03-21

**Authors:** Sawsan Abd Ellatif, Elsayed S. Abdel Razik, Marwa M. Abu-Serie, Ahmed Mahfouz, Abdullah F. Shater, Fayez M. Saleh, Mohamed M. Hassan, Walaa F. Alsanie, Abdullah Altalhi, Ghadir E. Daigham, Amira Y. Mahfouz

**Affiliations:** 1 Bioprocess Development Department, Genetic Engineering and Biotechnology Research Institute (GEBRI), City for Scientific Research and Technology Applications, New Borg El-Arab, Alexandria 21934, Egypt; sabdellatif@srtacity.sci.eg; 2 Plant Protection and Biomolecular Diagnosis Department, Arid Lands Cultivation Research Institute, City for Scientific Research and Technology Applications, New Borg El-Arab, Alexandria 21934, Egypt; eshabaan@srtacity.sci.eg; 3 Medical Biotechnology Department, Genetic Engineering and Biotechnology Research Institute (GEBRI), City of Scientific Research and Technology Applications, New Borg El-Arab, Alexandria 21934, Egypt; marwaelhedaia@yahoo.com; 4 National Health Service Foundation Trust (NHS), Manchester University, Manchester M14 5RH, UK; ahmed.mahfouz@mft.nhs.uk; 5 Department of Medical Laboratory Technology, Faculty of Applied Medical Sciences, University of Tabuk, Tabuk 71491, Saudi Arabia; ashater@ut.edu.sa; 6 Department of Medical Microbiology, Faculty of Medicine, University of Tabuk, Tabuk 71491, Saudi Arabia; fsaleh@ut.edu.sa; 7 Department of Biology, College of Science, Taif University, P.O. Box 11099, Taif 21944, Saudi Arabia; m.khyate@tu.edu.sa (M.M.H.); altalhi2001@hotmail.com (A.A.); 8 Department of Clinical Laboratory Sciences, The Faculty of Applied Medical Sciences, Taif University, P.O. Box 11099, Taif 21944, Saudi Arabia; w.alsanie@tu.edu.sa; 9 Centre of Biomedical Sciences Research (CBSR), Deanship of Scientific Research, Taif University, P.O. Box 11099, Taif 21944, Saudi Arabia; 10 Botany and Microbiology Department, Faculty of Science, Al-Azhar University (Girls Branch), Cairo 11651, Egypt; ghadirdaigham@azhar.edu.eg

**Keywords:** kefir grains, antibacterial activity, antifungal activity, gastric epithelial cells, probiotic, wound healing, anti-HCV activity, anti-HBV activity, immunomodulatory efficacy

## Abstract

The utilization of fermented foods with health-promoting properties is becoming more popular around the world. Consequently, kefir, a fermented milk beverage made from kefir grains, was shown in numerous studies to be a probiotic product providing significant health benefits. Herein, we assessed the antibacterial and antifungal potential of kefir against a variety of pathogenic bacteria and fungi. This study also showed the effectiveness of kefir in healing wounds in human gastric epithelial cells (GES-1) by (80.78%) compared with control (55.75%) within 48 h. The quantitative polymerase chain reaction (qPCR) results of kefir-treated HCV- or HBV- infected cells found that 200 µg/mL of kefir can eliminate 92.36% of HCV and 75.71% of HBV relative to the untreated infected cells, whereas 800 µg/mL (the highest concentration) completely eradicated HCV and HBV. Moreover, the estimated IC_50_ values of kefir, at which HCV and HBV were eradicated by 50%, were 63.84 ± 5.81 µg/mL and 224.02 ± 14.36 µg/mL, correspondingly. Kefir can significantly suppress the elevation of TNF-α and upregulate IL-10 and INF-γ in both treated HCV- and HBV-infected cells. High-performance liquid chromatography (HPLC) and gas chromatography-mass spectrometry (GC-MS) analysis of kefir revealed the presence of numerous active metabolites which mainly contribute to the antimicrobial, antiviral, and immunomodulatory activities. This study demonstrated, for the first time, the anti-HBV efficacy of kefir while also illustrating the immunomodulatory impact in the treated HBV-infected cells. Accordingly, kefir represents a potent antiviral agent against both viral hepatitis C and B, as well as having antimicrobial and wound healing potential.

## 1. Introduction

Over time, public interest in fermented dairy products and beverages featuring probiotics has grown in concert with the growing need for safe and high-quality health-food items, reducing the need for costly food supplements [1]. Among the informed health benefits, they are found to improve digestion and lactose tolerance, regulate plasma glucose concentration, minimize obesity’s consequences, improve blood cholesterol, and reduce cardiac and kidney hypertrophy, in addition to having antioxidant, antimicrobial, and intestinal bacteria modulation impacts [2,3]. Microbial cells have long been used as a renewable biofactory for the production of polysaccharides. Kefiran, one of the many appealing flexible biopolymers, has received a lot of attention in recent years because of its multiple nutritional benefits [4]. Kefir originated from the Caucasus Mountains’ northern side, where it has been consumed for thousands of years [5]. It is a traditional probiotic fermented milk liquor made from kefir grains that are tangy, lightly alcoholic, sour, with a creamy texture, and self-carbonated [6]. Kefir is metabolized by a diverse group of bacteria and yeasts, including *Lactobacillus, Lactococcus, Leuconostoc*, and *Acetobacter* species [5]. Furthermore, it has been shown to exhibit excellent antibacterial properties against a variety of foodborne pathogens, and also act as a defensive mechanism, insulating the immune system against viral infections [5,7,8,9]. Organic acids including lactic and acetic acid, as well as alcohol, are formed during fermentation and perform a biological role [10,11]. Previous findings have explored the health benefits of kefir, including antitumorigenic and anti-stress effects, and hypolipidemic actions [12,13]. Moreover, numerous studies have revealed kefir’s repressive actions versus food-borne Gram-negative and Gram-positive bacteria [13]. Kefir’s antibacterial force was verified versus many microbial pathogens [14]. Kefir has been demonstrated to accelerate wound healing, suppress tumor progression, and promote immune response changes to improve asthmatic symptoms and allergy [15]. ‘’Kefir and kefir byproducts as polysaccharides, proteins, peptides can restrict viral infection by altering the responses of the immune system and/or disrupting viral adherence. As a result, kefir itself with derivatives may be used to guard against viral infections’’ [16]. In addition, kefir exhibits anticancer, antifungal, and antibacterial characteristics [17], and also antimutagenic, epithelial-protective [18], anti-inflammatory [19], and antioxidant activity [20]. Kefir is a beneficial functional food used as an alternative therapy, according to the WHO, and research on it is of great interest [21]. Therefore, this prospective study was intended to monitor the antibacterial, antifungal, wound healing, and antiviral activities of kefir grains. Regarding the antiviral hepatitis assessment of kefir, this is the first study that investigated the anti-HBV activity of kefir. The antiviral assessment was associated with the determination of kefir’s effects on proinflammatory and anti-inflammatory cytokines (tumor necrosis factor (TNF)-α, interferon (INF)-γ, and interleukin (IL)-10).

## 2. Results and Discussion

In the current study, kefir was prepared under our investigational conditions. As documented by [22,23] the kefir grains or kefir beverages vary amongst countries since the milk used is either cow’s milk, goat milk, sheep milk, or camel milk. Moreover, Abd Ellatif et al. [24] reported the isolation of eight LAB from various dairy products including camel milk, yogurt, and cow milk.

### 2.1. Quantification of Protein and Phenolic Contents

Phenolic compounds are very important in defense mechanisms, such as anti-aging, anti-proliferative, anti-inflammatory, and antioxidant activities [25]. Polyphenols have a wide range of activities, including antioxidant, antimicrobial, wound healing, anti-HCV, anti-HBV, and immunomodulatory actions, according to several studies [26,27,28,29,30,31]. Consequently, it is critical to quantify and identify polyphenols in the kefir extract under study. Quantification of phenolics in kefir extracts by Folin–Ciocalteu technique revealed the existence of a decent quantity of total phenolics (4.5 ± 0.37 mg/g) calculated as per gallic acid for kefir extract, respectively. On the other hand, a total protein test was conducted to determine the protein content in kefir which is based on the concentration of starter and fermentation time. The result evoked that protein concentration was 1.06 ± 0.15 mg/g.

### 2.2. Quantification of Major Phenolic Compounds Using HPLC

**Figure 1:**
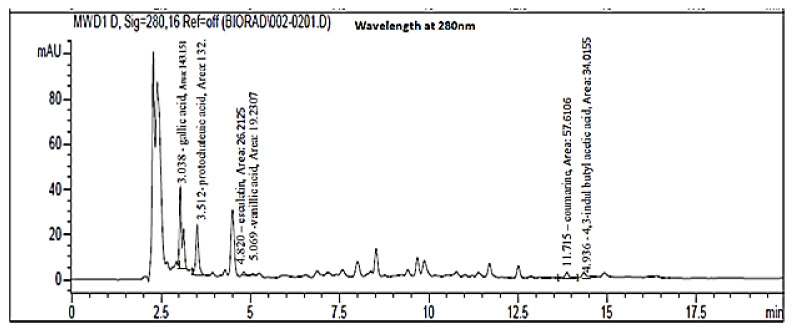
Detection of polyphenolic compounds of kefir by HPLC.

The results of the HPLC chromatogram of kefir polyphenolic compounds showed the presence of gallic acid, protocatechuic acid, vanillic acid, esculin, 3-indole butyl acetic acid, and coumarin (Figure 1 and Table 1). As shown in Table 1, the major polyphenolic constituents of kefir are gallic and protocatechuic acids. Govea-Salas et al., [32] found that gallic acid exhibits anti-HCV activity via diminished expression of NS5A-HCV and HCV-RNA. Gallic acid has a potent immunomodulatory effect by inhibiting the NF-KB signaling pathway (master for proinflammatory induction) [33]. Consequently, gallic acid could be one of the primary mediators of HCV activity of kefir. Besides the antioxidant and anti-inflammatory effects of protocatechuic acid [34], it revealed a strong anti-HBV activity. It was found that protocatechuic acid halted HBV replication by inhibiting HBV antigen secretion and activating the extracellular-signal-related kinase (ERK)1/2 pathway that downregulates hepatocyte nuclear factor (HNF) 4α/1 α [35]. Another previous study illustrated that protocatechuic acid enhanced the antiviral immune response (humoral and cellular) against the Newcastle Disease Virus and lowered viral load by improving the health of the infected chickens [36]. Accordingly, anti-HBV activity and the associated immunomodulatory effect in HBV-infected cells of kefir can be attributed mainly to the protocatechuic acid. Vanillic acid (V.A) a flavoring ingredient, is a benzoic acid derivative with a wide variety of biological activity, such as antioxidant, anti-inflammatory, and neuroprotective properties [37,38,39]. Vanillic acid, an oxidized form of vanillin also known as (4-hydroxy-3-methoxy benzoic acid), is one of the most well-studied polyphenolics used for therapeutic characteristics [40]. Vanillic acid has been proven in numerous studies to bear a variety of pharmacological effects, including antihypertensive, antioxidant, and anti-inflammatory properties [39,40]. Moreover, vanillic acid was reported to have an anti-filarial, antimicrobial impact in addition to efficiency in inhibition of snake venom activity [41]. Additionally, the potent neuroprotective effect of vanillic acid against lipopolysaccharides (LPS) -induced neurotoxicity in mice brains was recorded by [37]. Another study declared that vanillic acid reduced the cytopathic effect of RNA double-stranded viruses (rotavirus) and exhibited antimicrobial activity against carbapenem-resistant *Enterobacter cloacae* (CREC) [42].

In vitro, esculetin, a coumarin molecule was found to decrease the expression of not only HBV surface antigen (HBsAg) and HBV core antigen (HBeAg), but also HBV DNA and HBV pleiotropic regulatory protein X (HBx). Moreover, esculetin has been shown to inhibit HBV replication in vivo, reducing the activity of alanine aminotransferase (ALT) and aspartate aminotransferase (AST), and also reducing the activity of alanine aminotransferase (ALT) and aspartate aminotransferase (AST) [43]. Esculetin effectively inhibits HBV replication in vitro and in vivo, suggesting that it could be developed further as an antiviral medication. Only one study has shown that one of the coumarin derivatives can suppress HBV replication and lower the level of HBV-cc DNA in HBV-infected cells [44]. Biological actions of derivatives containing the indole core include antidiabetic, anticancer, antibacterial, anti-HIV, antiviral, anti-inflammatory, and antioxidant. Indole has a broad range of biological functions and offers a lot of potential when it comes to novel therapeutic options [45].

### 2.3. Gas Chromatography/Mass Spectrometry Analysis (GC/MS) of Kefir Extract

**Figure 2:**
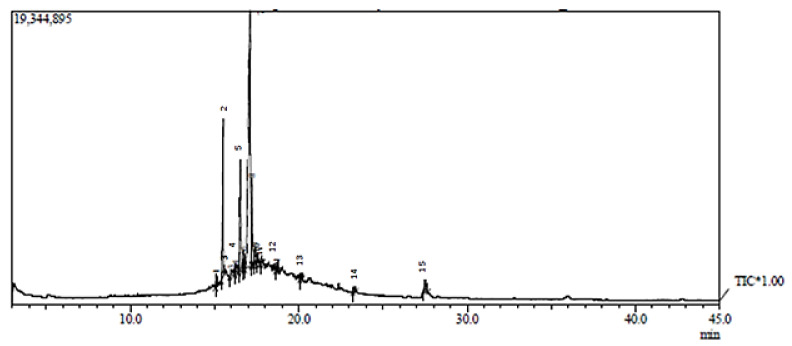
GC-MS chromatogram of kefir extract.

In the current study, the bioactive components in kefir were identified via GC-MS analysis (Figure 2). The compound’s names and classes, with corresponding peaks at different retention times in addition to molecular formula, molecular weight, and biological activities of kefir extract were represented in Table 2. GC-MS analysis revealed the presence of 14 metabolites in the tested extract. The main compounds in the kefir are Hexadecanoic acid, methyl ester, n-Hexadecanoic acid, Heneicosane, Hexadecane, 2,6,10,14-tetramethyl, 9-Octadecenoic acid, methyl ester, (E)-, Octadecanoic acid, methyl ester, cis-10-Heptadecenoic acid, Octadecanoic acid, Octacosane, Eicosane, Pentadecane, 8-hexy Nonacosane, Triacontane, 1,2-Benzenedicarboxylic acid, mono (2-Ethylhexyl) ester; 9-Octadecenoic acid (Z)-, and 2,3-dihydroxy propyl ester. The GC-MS analyses showed numerous anticancer compounds present in the extract of kefir. A study conducted by [46] evoked that Hexadecanoic acid (palmitic acid) and Octadecanoic acid are saturated long-chain fatty acids exhibiting antimicrobial action and selective cytotoxicity against human leukemic cells, as well as in vivo antitumor activity in mice. Moreover, Palmitic acid has been demonstrated to be a highly antimutagenic constituent of milk fat, with higher quantities in kefir than in milk or yogurt. According to the study of Vieira et al., [47] the highest concentration of palmitic acid in fermented milk may boost the anti-mutagenic activity. Stored kefir contained higher amounts of oleic acid and monounsaturated fats and lower levels of saturated fatty acids, raising the chance of antimutagenicity and anticarcinogenicity in stored milk and possibly improving the nutritive value of fatty acids when compared to fermented kefir. Esters and fatty acid esters obtained through our results in the form of (Hexadecanoic acid methyl ester, 9-Octadecenoic acid methyl ester (E), 1,2-Benzenedicarboxylic acid, mono(2-ethylhexyl) ester, and 9-Octadecenoic acid (Z)-, 2,3-dihydroxy propyl ester) are positive charges and hydrophobic in general; this hydrophobicity facilitates electrostatic interactions with bacterial cell components, resulting in cell viability loss due to the production of fully deenergized dead cells. They additionally serve as surfactants, inhibiting the growth of foodborne pathogens [23,48]. More interestingly, Cis-10-Heptadecenoic acid, (monounsaturated fatty acid) obtained in our GC mass results has potential antitumor activity and was reported to impede HL-60 cell propagation [49]. Besides, (Octacosane and Eicosane) obtained here bear antimicrobial efficacy, potent antioxidant, and anti-inflammatory action. In brief, the GC-MS-analyses revealed the existence of many bioactive metabolites in kefir extract which exhibit an extensive spectrum of biological activities such as anti-inflammatory, antibacterial, antioxidant, and anticancer effects, which are relevant to our investigation as documented in Table 2. The GC-MS analysis provides a typical spectrum output for each chemical detected in the examined sample. As a result, in recent years, GC-MS has been considered a major technology platform for characterizing secondary metabolites in both plant and non-plant species [50,51].

### 2.4. Recognition of Kefir Antimicrobial Activity

**Figure 3:**
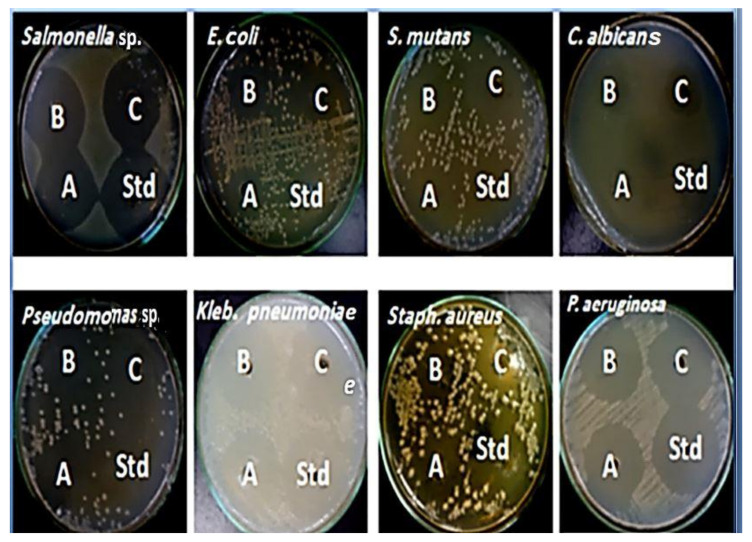
Antimicrobial activity of kefir grain powder: (**A**): 40 µg/mL, (**B**): 50 µg/mL and (**C**): 60 µg/mL against selected pathogens compared with 10 µg/mL ampicillin in term of a zone of inhibition (ZOI).

Kefir’s medicinal and biological restorative characteristics are well-known. In the current investigation, the antimicrobial activity of kefir was assessed, and the kefir showed excellent antibacterial activity versus all tested microbes (Figure 3). The results disclosed that 60 µg/mL of kefir displayed the greatest action against *Klebsiella Pneumoniae*, *Pseudomonas* sp., *Streptococcus* *mutans*, and *Salmonella* sp. with a zone of inhibition (ZOI) about 43.2 mm, 39.7 mm, 35.1 mm, and 33.1 mm, respectively (Table 3). Furthermore, the kefir extract exhibited antifungal properties that are highly effective against *Candida albicans* with a ZOI of about 23.0 mm (Table 4). Similar reports have explained the ability of lactic acid bacteria (LAB) to develop bacteriocins that inhibit food-poisoning bacteria *Listeria monocytogenes* [35].

Kefir has a variety of LAB species that are recognized for producing bacteriocin and providing probiotic benefits [3,66]. Our findings are consistent with previous studies [67,68] that reported the antibacterial activity of kefir against selected foodborne pathogens. The results gained from some studies showed that the microorganisms from kefir products hindered the growth of *Escherichia coli*, *Listeria* *monocytogenes, Staphylococcus aureus, Shigella* spp., *Salmonella* spp., *Bacillus cereus, Bacillus. subtilis, Klebsiella pneumoniae, Pseudomonas aeruginosa*, and *Enterococcus faecalis* [10,19,69,70]. A study of [23] evoked the superior antimicrobial activity of kefir beverage against *E. coli* ATCC11229, *L. monocytogenes* ATCC 4957, *B. cereus* ATCC 14579, *S. typhimurium* ATCC 14028 as well as *A. flavus* ATCC 16872 and *A. niger* ATCC 20611.

**Figure 4:**
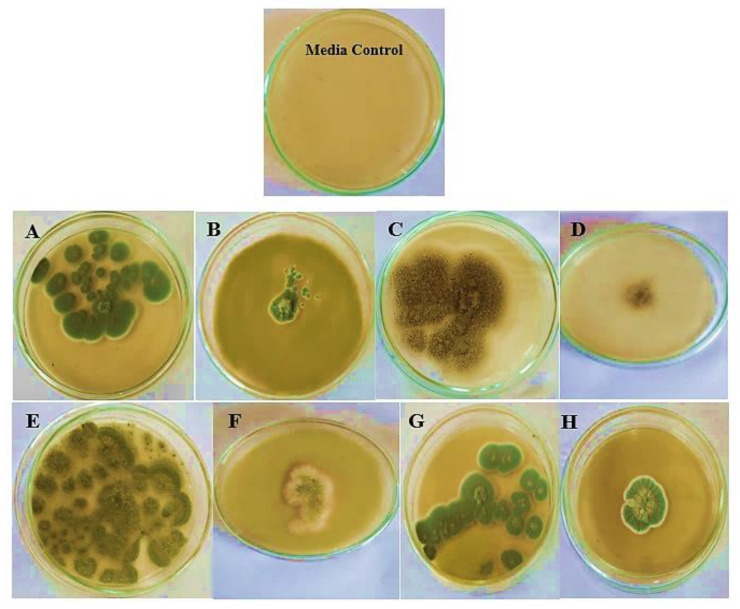
Antifungal potential of kefir extract (100 mg/mL) against selected pathogenic fungal strains: (**A**): *Aspergillus fumigatus* control, (**B**): *Aspergillus fumigatus* + kefir extract, (**C**): *Aspergillus brasiliensis* control, (**D**): *Aspergillus brasiliensis* + kefir extract, (**E**): *Aspergillus flavus control, (**F**): Aspergillus flavus* + kefir extract, (**G**): *Penicillium expansum* control, (**H**): *Penicillium expansum* + kefir extract.

It was deduced from the data listed in Table 4 and Figure 4 that kefir extract showed superior antifungal efficacy against all of the investigated fungi. As reported by previous studies [10,51], kefirs from various sources revealed numerous antifungal spectra which validates our findings. In another study, kefir showed inhibitory potential toward all tested strains, except for *P. expansum* LPH6 [50]^.^ Additionally, previous findings confirmed that kefir prevented the growth of *Aspergillus carbonarius A. niger, Aspergillus parasiticus, Aspergillus fumigatus, Aspergillus flavus, Penicillium* spp, *Trichoderma longibrachiatum*, and *Rhizopus microspores* [23,71,72,73].

### 2.5. Determination of Kefir Wound Healing Activity

**Figure 5:**
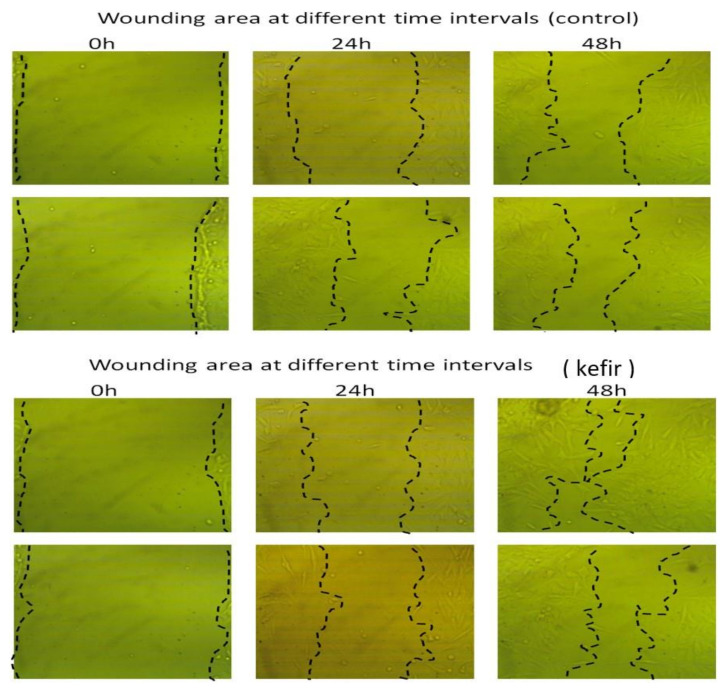
Wound-healing activities of kefir extract on human gastric epithelial cells (GES-1).

**Figure 6:**
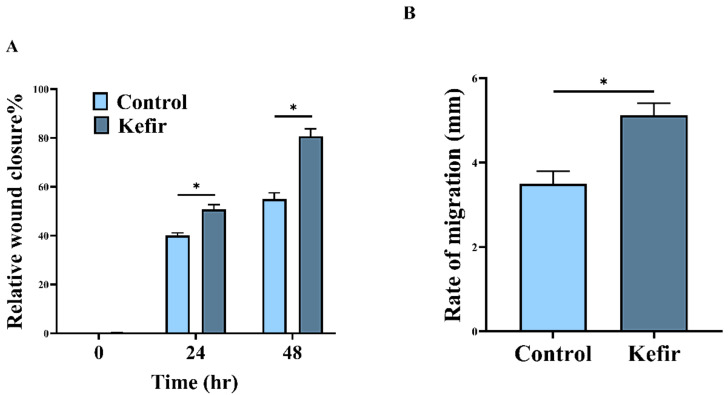
Wound healing assay (**A**): Determination of relative wound closure in GES-1 cells treated with kefir extract compared to control. The wound-healing test was achieved at 0, 24 and 48 h. in GES-1 cells treated with kefir extract. Relative wound closure was concluded by measurement of the wounds width (**B**): Rate of cell migration. All data were expressed as mean± SEM. The data mean is considered significantly different at *p ≤* 0.05 and is marked as *.

In an attempt to evaluate the cellular repair proportion and consequently wound-healing activities of kefir, human gastric epithelial cells (GES-1) were scratched using scratch wound assay and then treated with kefir extract at different periods. The results in Figure 5 revealed that the cell-free regions were virtually completely enclosed by 80.78% compared with the untreated control (55.75%) within 48 h. Moreover, (Figure 6A) determines the relative wound closure in GES-1 cells treated with kefir extract compared to control while, (Figure 6B) illustrates the rate of cell migration.

The obtained results are in perfect harmony with the results obtained by [74] who indicated that the cell-free areas were nearly entirely enclosed later than 64 h from the assay beginning. Wound healing is a complicated process of mending cell structure and extracellular constituents following tissue injury. The scratching assay is a basic in vitro approach for studying directed cell migration in wound-healing procedures. Moreover, it was revealed that scratch wound assay represents a widely used strategy to assess cellular repair proportion, enabling the analysis of cell migration, tissue restructuring, as well as cell division [74]. It is feasible to evaluate the rate of wound closure and examine cell–cell and cell–matrix interactions by quantifying the repair of the scarred region. The summation of many cell processes, such as cell migration, multiplication, and morphological change, in relation to both soluble and rigid substrates factors, controls the rate of “wound” closing. When a 70% kefir gel was applied to a wound inoculated with *S. aureus*, it was found to be more effective at healing and scar formation than that gained by the neomycin-clostebol as a positive control [19,75]. Since wounds are fundamentally linked with changes in the local microbiota as a result of injury and immune response activation, using topical probiotics to prevent infection, manage inflammation, and possibly boost healing could be beneficial [76]. Additionally, another study of [77] concluded that the use of kefir gel treatment improved the healing of experimental burn injury by lowering inflammation and enhancing epithelization, making it a successful therapeutic approach.

### 2.6. Determination of Kefir Anti-HCV and Anti-HBV Activities

**Figure 7:**
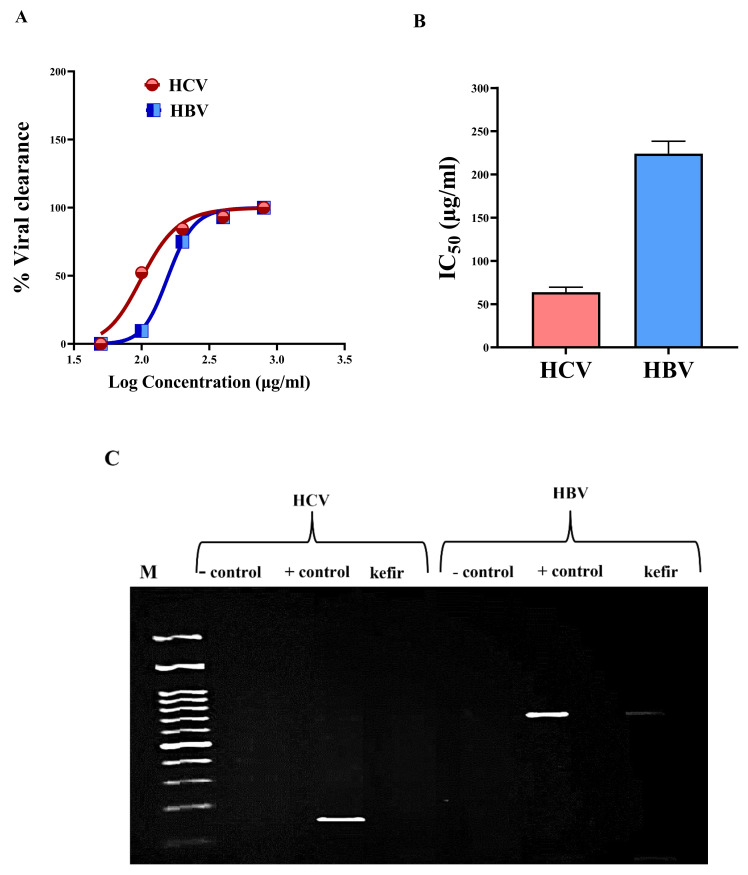
Antiviral activity of kefir. (**A**) Dose–response curve of anti-HCV and anti-HBV activities of kefir extract. (**B**) The estimated IC_50_ of kefir extract against HCV and HBV. All data were demonstrated as mean ± SEM. (**C**) Gel electrophoresis images of nested PCR for viral hepatitis-infected cells after treatment with kefir extract (400 µg/mL) compared to the negative (−) and positive (+) controls of viral-infected PBMCs.

For investigating the antiviral hepatitis activity of kefir milk extract, the safe dose (EC_100_ 1041.2 ± 1.77 µg/mL) was detected initially, to use without affecting the viability of viral host cells and to avoid any false factors interfering with the resulting antiviral effect. According to TaqMan quantitative polymerase chain reaction (qPCR) qPCR, 200 µg/mL of kefir extract can eliminate 92.36% of HCV and 75.71% of HBV (Figure 7A). Both viruses were mostly declared (≥92%) at 400 µg/mL of kefir extract. At the highest used concentration (800 µg/mL), HCV and HBV were completely eradicated in the aqueous extract-treated cells (Figure 7A). Figure 7B demonstrated that IC_50_ values of kefir were 63.84 ± 5.81 µg/mL and 224.02 ± 14.36 µg/mL against HCV and HBV, respectively. The IC_50_ for the anti-HCV effect was lower than that of anti-HBV indicating that kefir was more effective against HCV than HBV (Figure 7B). Furthermore, this higher anti-viral efficacy of kefir was verified by a gel image of nested PCR (Figure 7C) that illustrated the absence of positive HCV band and very faint positive HBV band in kefir (400 µg/mL)-treated PBMCs.

**Figure 8:**
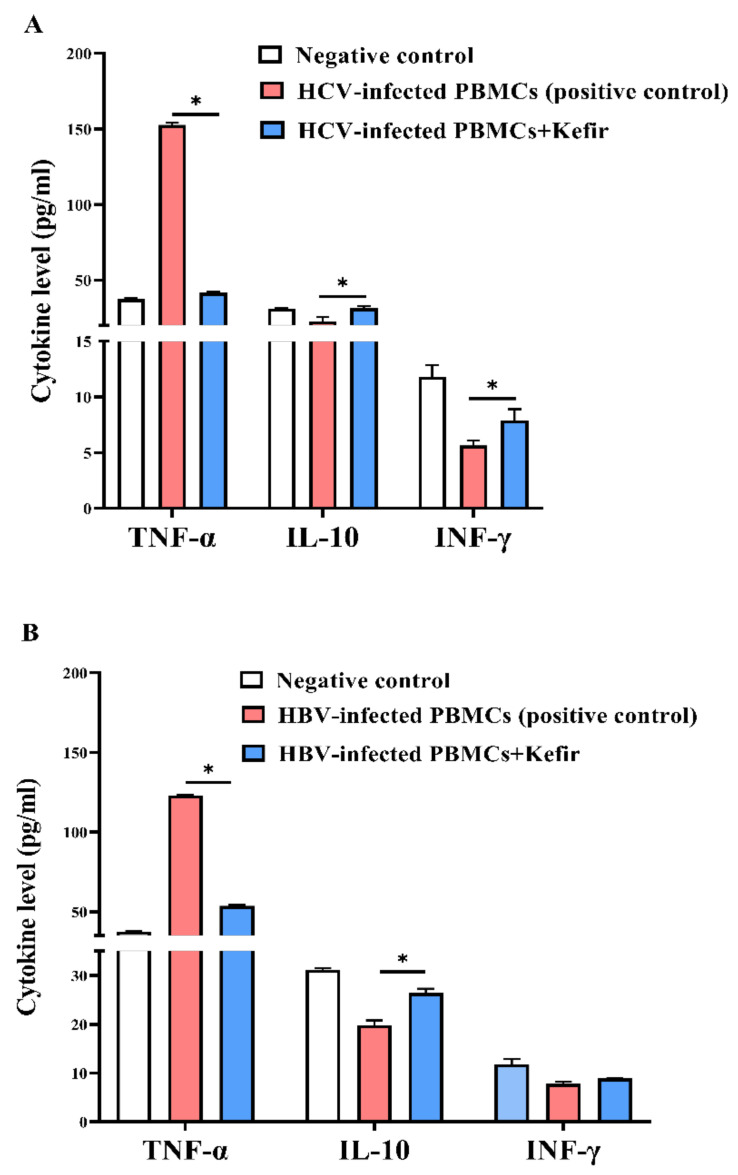
Cytokine levels (pg./mL) of TNF-α, IL-10, and INF-γ after treatment of infected PBMCs with kefir extract in comparison with negative and positive controls. (**A**) HCV and (**B**) HBV. All data are demonstrated as mean ± SEM. The data mean is considered significantly different at *p* ≤ 0.05 and is marked as *.

HCV and HBV infections represent major health issues that affect people all over the world [27], particularly Egypt where it holds the greatest hepatitis C virus prevalence [78]. According to the WHO, there are 58 million and 296 million people infected with HCV and HBV, respectively, showing that HBV infectivity is 5 times that of HCV [79]. As a result of the high cost of medications, particularly in low-income countries with the highest HCV and HBV prevalence, it is preferable to look for low-cost and efficient alternatives for effective treatment for both hepatitis viruses. In this concern, multiple studies have proven the beneficial effects of probiotic strains on preventing a wide range of dangerous liver disorders [80]. A previous study revealed that kefir reduced viral load, improved liver function, and improved lipid profile in HCV patients. This antiviral effect may be attributed to the antioxidant, immunostimulant, and anti-inflammatory activities of kefir [66]. Based on our best knowledge this is the first study that declared the anti-HBV efficacy of kefir (Figure 7) with illustrating the aforementioned immunomodulatory effect in HBV-infected cells (Figure 8).

Additionally, (Figure 8A) reveals that kefir can significantly suppress the elevation in TNF-α by 72.72% and enhance the secretion of IL-10 and INF-γ by 1.38- and 1.4-fold, respectively, compared to untreated HCV-infected PBMCs. In addition, as compared to untreated HBV-infected PBMCs, this extract of milk kefir dramatically lowered TNF-α by 56.40% (*p* < 0.05) and promoted IL-10 and INF-γ secretion by 1.33-fold and 1.15-fold, respectively (Figure 8B). As stated by a recent study, IFN-γ is a cytokine that performs a crucial function in stimulating and regulating an assortment of immune responses [81].

IFN-γ is a multifunctional cytokine with antiviral, anticancer, and anti-inflammatory properties. As a result, it coordinates both the innate and adaptive immune responses [82]. IFN-γ activates the immune system response and boosts pathogen clearance in an inflammatory situation; moreover, it inhibits over-activation of the immune response and tissue injury [83]. Rises in the serum concentration of IFN-γ have been detected in retort to vaccination against smallpox, indicating a parallel mechanism of action of kefir to that highlighted directly above [84]. It was pointed out that kefir intake caused a 42% decrease of TNF-α/IL-10 manifestation and a 50% decline in proinflammatory IL-6 expression in accord with a boosting anti-inflammatory IL-10 expression [85]. More interestingly, a previous study indicated that kefir could lower the release of pro-inflammatory molecules linked to the mucosal immune response [86]. Moreover, it was reported that kefir exhibited anti-inflammatory impacts on colitis and cancer via reducing the construction of pro-inflammatory mediators (TNF-α and IL-1) and raising anti-inflammatory cytokines [87,88]. The effect of a kefir-derived antimicrobial component on Zika virus cytopathic consequences, lymphocyte proliferation, and reduction of TNF-α/IL-10 and IL-6/IL-10 levels after kefir administration was explored previously [85]. More importantly, the potential immunomodulatory effect of kefir as a potent suppressor of TNF-α, IL-6, and IL-1 and enhancer of CD8^+^ T cells, immunoglobulin (Ig)G^+^B cells with boosting the production of IL-2 and IFN-γ could be a promising therapeutic agent against cytokine storm in COVID-19 patients [89].

## 3. Materials and Methods

### 3.1. Chemicals and Reagents

The chemicals, solvents, and media used in the study were of standard analytical grade and were purchased from Sigma-Aldrich, inc., St. Louis, MO, USA. Cell culture reagents were obtained from GIBCO, Brooklyn, NY, USA. All molecular reagents were purchased from Thermo Scientific, Waltham, MA, USA except for the viral DNA extraction kit which was obtained from Qiagen, Hilden, Germany.

### 3.2. Cultivation and Extraction of Milk Kefir

#### Kefir Production

In this regard, 0.01 U freeze-dried, aromatic kefir culture PROBATE KC3 (Danisco, Denmark) was used as a starter culture to ferment 1 L of milk. A standard, freeze-dried culture was applied instead of using traditional kefir grain. The starter culture was composed of *Lactococcus lactis subsp.* *lactis, Lactococcus lactis subsp. cremoris, Lactococcus* *lactis subsp. diacetylactis, Leuconostoc mesenteroides* *subsp. cremoris, Lentilactobacillus kefiri, Kluyveromyces marxianus var. marxianus,* and *Saccharomyces* *unisporus.* Camel milk was employed in this study to increase microbiological quality. The fermented kefir milk was prepared according to the method of [90] with slight modifications. Briefly, the raw camel milk was pasteurized at 90 °C for 30 min in a water bath and cooled to about 30 °C. Next, the heat-treated milk was inoculated with 2% kefir grains and incubated at room temperature for 18 h. At the end of the fermentation process, the grains and milk were separated using a sterilized cheesecloth filter (2 mm pore size) then the kefir sample was stored in the refrigerator at 4 °C to determine the total phenolic content, antibacterial, antifungal, antiviral, antioxidant, and wound healing activities.

### 3.3. Quantification of Protein and Phenolic Contents

Bradford Coomassie’s Brilliant Blue assay was used to determine the protein level of aqueous and ethanolic kefir extracts [91]. The generated, blue-colored intricate was detected at 595 nm. Next, the protein content was calculated using the bovine serum albumin standard curve. By using Folin–Ciocalteu reagent and gallic acid as a standard, the phenolic content of each extract was evaluated [34].

#### Quantification of Major Phenolic Compounds Using HPLC

The phenolic compounds in the kefir sample were assessed according to the method described by [92]. The frozen kefir extract was thawed and centrifuged for 10 min at 12,000 rpm. Next, A10 μL of the extract was injected into an Agilent 1100 Infinity Series HPLC system equipped with a diode array detector (SPD-M10Avp) for quantitative analysis. Phenomenex Luna C-18(2) column (4.6 mm ID × 25 cm, 5 mL.) was used for the analysis. The mobile phase was a mixture of 0.1% orthophosphoric acid (A) and acetonitrile (B). The gradient used was: 0–12 min, 15% B; 12–22 min, 25% B; 22–30 min, 15% B. The flow rate and column temperature were maintained as 1.0 mL/min and 35 °C, respectively. Polyphenol standards: Gallic acid, catechin, coumaric acid, Esculetin, Tannic acid, caffeic acid, quercetin, cinnamic acid, dihydroxybenzoic acid, 3-indole butyl acetic acid Guaiacol, and Protocatechuic acid were obtained from Sigma–Aldrich. Acetonitrile (HPLC), methanol (HPLC), acetic acid (HPLC), orthophosphoric acid, and ethanol were obtained from Merck (Darmstadt, Germany). A stock standard solution (100 μg/mL) of each phenolic compound was prepared in methanol by weighing up about 0.0050 g of the analyte into a 50 mL volumetric flask. All standard solutions were kept in the dark at 5 °C. The calibration curves of the standards were prepared by serial dilution of the stock standards (five sets of standard dilutions) with methanol to produce 5, 10, 20, 40, and 80 μg/mL for each standard. Kefir polyphenols were detected at 280 nm. The resolution peaks have been detailed on the HPLC chart corresponding to the retention time of each compound. The results were expressed in microgram per gram dry weight (µg/mL) of the fermented kefir sample.

### 3.4. Gas Chromatography/Mass Spectrometry Analysis (GC/MS) of Kefir Extract

Identification of the chemical moieties of kefir extract was performed by gas chromatography combined with the mass spectrometry technique. The GC–MS analysis was carried out in a Shimadzu GC-MS-QP 2010 Plus fitted with an RTX-5 (60 mmL × 9 0.25 mml, D., 9 0.25 µm) capillary column in JNU, New Delhi, Restek capillary column using helium as a carrier at 300 °C. The oven temperature program was initiated at 50 °C, held for 3 min, then increased at rate of 8 °C to 250 °C min^−1^ and held for 10 min. The spectrophotometer was operated in electron impact mode. The injector, interface, and ion source were kept at 250 °C, 230 °C, and 220 °C, respectively. Split injection (1 µL diluted sample in n-hexane (1:1 *v*/*v*) injected) was conducted with a split ratio of 1:20 and column flow of 1.5 mL/min. The identification of the components of kefir was based on a comparison of their relative indices and mass spectra by computer matching with WILEY and National Institute of Standards and Technology (NIST08) libraries data (http://webbook.nist.gov accessed on 2 November 2021) provided with the computer controlling GC–MS system. Individual isolated compound identifications were also performed by comparing their mass spectra and retention times with authentic compounds and literature data [93]. This step was carried out at a special unit for scientific Services and Technology, the city of scientific research and Biotechnological applications, New Borg El-Arab City, Alexandria, Egypt.

### 3.5. Recognition of Kefir Antimicrobial Activity

#### 3.5.1. Test Organisms

*Salmonella* sp., *E. coli* ATCC 25922, *Streptococcus mutans* ATCC25175, *Pseudomonas* sp., Klebsiella pneumoniae ATCC 13883, *Staphylococcus aureus* EMCC1351 and Candida albicans ATCC10231 as well as *Aspergillus fumigatus*, and *Penicillium expansum* were kindly obtained from Bioprocess Development Department, Genetic Engineering and Biotechnology Research Institute (GEBRI), the City of Scientific Research and Technological Applications, New Borg El-Arab, 21934, Alexandria, Egypt. *Aspergillus flavus* AUMC 13922 and *Aspergillus brasiliensis* AUMC 13921 were obtained from the Botany and Microbiology Department, Faculty of Science, Al-Azhar University (Girls Branch), Cairo, Egypt.

#### 3.5.2. Assay of Antibacterial Activity of Kefir

Antibacterial action of kefir grains extract was appraised using the well-cut diffusion method as described. Cell suspensions of 4 × 10^5^ CFU/mL of 7 bacterial strains (*Salmonella.* sp., *E. coli* ATCC 25922, *Streptococcus mutans* ATCC25175, *Pseudomonas.* sp., *Klebsiella. pneumoniae* ATCC 13883, *S. aureus* EMCC1351) and pathogenic fungus *C. albicans* ATCC10231) were prepared from organisms grown in LB agar media at 35 °C for 24 h. Disks were applied to the agar surface previously inoculated with 100 µL organism suspension. Antibiotics (10 μg/mL, ampicillin) were used to compare the antimicrobial activity, and kefir extracts (40 μg/mL, 50 μg/mL, and 60 μg/mL were pipette onto 5 mm diameter well in plate agar). Incubation of the inoculated plates was achieved at 35 °C for 24 h and then the resulted growth inhibition zones were quantified. All treatments were performed in triplicates and mean values of results were utilized.

#### 3.5.3. Assay of Kefir Antifungal Efficacy

Kefir antifungal activity was carried out following the method previously described by [94] with minor adjustments. The fungal strains were sub-cultured on potato dextrose agar slant for 7 days. The agar slant of each fungal strain was flooded with 10 mL of sterile sodium lauryl sulfate (0.01% *w*/*v* in NaCl 1%) to acquire monophonic spore suspension. Filtration of the suspensions through Whatman No.1 paper and counting of the conidia using a Neubauer chamber was performed. The fungal inoculum was amended into 5 × 10^5^ conidia/mL. According to [95] method, the sterilized malt agar medium was mixed after cooling to about 45 °C with (10% *v*/*v*) of kefir extract (100 mg/mL). Next, 20 mL of the agar medium was poured into 12 mm diameter Petri dishes. Inoculation by 10 μL of the spore suspensions containing 5 × 10^5^ spore/mL, in the middle of the medium was performed. Agar media plate inoculated with a modest amount of fungal culture without any kefir extract was served as the positive control. Incubation of all plates was conducted at 28 ± 1 °C for 7 days. After ending the incubation period, each colony’s average diameter was measured to determine the diameter of the growth inhibition zone (ZOI).

### 3.6. Determination of Kefir Wound Healing Activity by Scratch Assay

This assay is conducted by scratching the adherent cell monolayer with a pipette tip or a syringe needle to produce an artificial damage region surrounding the cells. From wound initiation until wound repair with cell confluence, photographic pictures of cell migration were captured over time [96]. In the current study, human gastric epithelial cells (GES-1) were acquired from Cancer Institute, Cairo, Egypt. GES-1 cells were cultivated in 6 multi-well plates until confluent as described by [97]. A sterilized yellow pipette edge held at a 30-degree angle (to maintain restricted scratch size) was used to make a straight scratch and subjected to treatment with kefir extract for 48 h. Next, imaging of both wound edges using the 10× objective was performed. The data were stated as mean ± SEM and variance, and *p* ≤ 0.05 was counted as a statistical consequence.

### 3.7. Determination of anti-HCV and Anti-HBV Activities

#### 3.7.1. Detection of Cytotoxicity of Milk Kefir Extracts against Viral Host Cells

Human peripheral blood mononuclear cells (PBMCs) were isolated using the Ficoll-Hypaque density gradient centrifugation method. Briefly, healthy volunteers’ blood samples were carefully placed on Ficoll-Hypaque and centrifuged at 2000 rpm for 30 min at 25 °C. The PBMC layer was then collected, mixed in RPMI-1640 media, and spun for 10 min at 1650 rpm. Finally, cells were mixed with RPMI-1640 medium comprising 10% fetal bovine serum (FBS) and counted employing trypan blue exclusion method for seeding 1 × 10^5^ cells/well in 96-well culture plate.

Serial concentrations of kefir extract were incubated with PBMCs for 72 h in a 5% CO_2_ incubator. Then, 20 µL of MTT solution (5 mg/mL) was added and incubated for 4 h in a 5% CO_2_ incubator [98]. Then MTT solution was discarded, 100 µL of 100% DMSO was added and mixed for 10 min. The O.D. was determined at 570 nm via a microplate reader (BMG LabTech, Ortenberg, Germany) to estimate the safe dose (EC_100_) of each extract that causes 100% cell viability using GraphPad instant.

#### 3.7.2. Quantitative Detection of Anti-HCV and Anti-HBV Activities of Kefir Milk

About 1 × 10^6^ PBMCs were seeded per well in a 12-well plate then RPMI medium containing HCV (2.9 × 10^5^ copies/mL, genotype 4a)—or HBV (1 × 10^5^ copies/mL) -infected serum was added to all except negative control wells containing RPMI medium with 10% FBS. After 2 h incubation in a 5% CO_2_ incubator, the infected medium was substituted with a fresh RPMI medium comprising 10% FBS, and plates were incubated overnight in a 5% CO_2_ incubator. Then, different concentrations of kefir extract (50, 100, 200, 400, and 800 µg/mL) were incubated with the infected HCV-and HBV-infected cells, separately, for 72 h in a 5% CO_2_ incubator. The negative uninfected control, positive infected control, and treated cells were collected, separately, to quantify viral load using the Cobas AmpliPrep instrument and the Cobas TaqMan analyzer (CAP-CTM). The dose (IC_50_) of kefir at which viral load decreased by 50% was calculated using GraphPad instant.

The effective anti-viral dose (400 µg/mL) of kefir extract was confirmed, against HCV and HBV, by nested PCR. For HCV, the total RNA was extracted from negative and positive controls as well as active kefir extract-treated cells using Gene JET RNA purification Kit (Thermo Scientific, Waltham, MA, USA). The complementary DNA and the first PCR reaction of the nested PCR were performed using the Ready-To-Go RT-PCR beads (Amersham Pharmacia Biotech, Piscataway, NJ, USA) and primers. These primers included 1CH; 5′-GGTGCACGGTCTACGAGACCTC-3′, 2CH; 5′-AACTACTGTCTTCACGCAGAA-3′ and P2; 5′-TGCTCATGGTGCACGGTCTA-3′. Meanwhile, the second PCR reaction of the nested PCR was performed using Taq PCR master mix and primers (D2; 5′-ACTCGGCTAGCAGTCTCGCG-3′ and F2; 5′-GTGCAG CCTCCAGGACCC-3′) [32]. Regarding HBV, viral DNA was extracted using a viral DNA extraction kit. Then, the nested PCR was carried out according to [99]. For the first PCR, the primer sequences are 5′-AAGCTCTGCTAGATCCCAGAGT-3′ and 5′-CATACTTTCCAATCAATAGG-3′, while for the second reaction, the primer sequences are 5′-TGCTGCTATGCCTCATCTTC-3′ and 5′-CATACTTTCCAATCAATAGG-3′. Finally, on 2% agarose, PCR products were purified, and the HCV band was visualized at 174 and 681 base pair (bp), respectively, using a UV transilluminator.

#### 3.7.3. Determination of Cytokine Levels in Kefir Extract-Treated Viral-Infected PBMCs

After infecting PBMCs with HCV or HBV-infected serum and treating with the most active kefir extract concentration (400 µg/mL), supernatants were collected after centrifuging the cells. Cytokines (including, TNF-α, INF-γ, and IL-10) were measured in the collected supernatant according to manufacturers of RayBio^®^ human ELISA Kits.

### 3.8. Statistical Analysis

All data were expressed as mean ± standard error (SEM) and an unpaired T-test was utilized for comparison using SPSS 16 software. Statistical differences were illustrated at *p*-value *≤* 0.05.

## 4. Conclusions

In conclusion, kefir has been connected with health grants for decades. Milk kefir is a conventional product of fermented milk whose consumption is progressively becoming more popular. Comprehensive and in-depth laboratory analysis of the antibacterial, antifungal, and antiviral effectiveness of kefir was carried out in the present study. The study highlights the potential wound healing activity of kefir on GES-1 cells. As stated from previous reports, one supposed key strategy to safeguard patients from the cytokine storm is to impede the work of interleukins or to offer specific compounds to stifle inflammation. In our study, kefir exerted a potent inhibitory upshot of proinflammatory cytokines with stimulation of anti-inflammatory cytokines in treated HCV-and HBV-infected cells. Hence, this study discovered the promising anti-HBV activity and immunomodulatory effect of kefir in treated HBV-infected cells. According to these current findings, kefir is an effective anti-HBV agent with an ameliorating antiviral immune response in addition to bearing anti-HCV, antibacterial, anti-fungal, and wound healing activities.

## Figures and Tables

**Table 1:** Polyphenol’s concentration of kefir.

Sample	Active Components	Peak Area	Conc. (µg/mL)	Retention Time	Units
**Kefir**	Gallic Acid	6.048	8.6543 ± 0.00321	3.038	µg/mL
	Protocatechuic Acid	8.20	10.849 ± 0.00153	3.512	µg/mL
	Esculatin	9.719	2.545 ± 0.00265	4.820	µg/mL
	Vanillic Acid	5.54	1.066 ± 0.01000	5.069	µg/mL
	Coumarin	2.41	1.390 ± 0.01000	11.715	µg/mL
	4,3-Indole Butyl Acetic Acid	9.527	3.240 ± 0.02000	14.936	µg/mL

All values were expressed as mean± SEM (*n* = 3).

**Table 2:** Identified compounds in the kefir extracts using GC-MS chromatogram.

ID	Name	R. Time	Base Beak (*m*/*z*)	Area	Height	Mol. Formula and mol. wt.	Ret. Index	Reported Bioactivity
**1**	**Hexadecanoic acid methyl ester**	15.093	74.00	620,892	168431	C_17_H_34_O_2_ (270)	1878	Antioxidant, decrease blood cholesterol, anti-inflammatory [52].
**2**	**n-Hexadecanoic acid**	15.525	43.00	3,772,458	956337	C_16_H_32_O_2_ (256)	1968	Antibacterial and antifungal [53]
**3**	**Heneicosane**	15.998	57.00	397,344	75000	C_21_H_44_ (296)	2109	Microbicide activities [54]
**4**	**Hexadecane, 2,6,10,14-tetramethyl**	16.259	57.00	430,571	96464	C_20_H_42_ (282)	1753	Antifungal compound [55,56]
**5**	**9-Octadecenoic acid methyl ester (E)-**	16.542	55.00	1,989,019	523430	C_19_H_36_O_2_ (296)	2085	Anti-inflammatory, antiandrogenic, cancer preventive, dermatitigenic, irritant, anti-leukotriene—D4, hypocholesterolemic, 5-alpha reductase inhibitor, anemia genic, insectifuge, flavor [57]
**6**	**Octadecanoic acid** **methyl ester**	16.735	74.00	578,657	233210	C_19_H_38_O_2_ (298)	2077	Antifungal and antioxidant [58]
**7**	**cis-10-Heptadecenoic acid**	17.122	55.00	11,457,870	1249206	C_17_H_32_O_2_ (268)	2075	-
**8**	**Octadecanoic acid**	17.225	43.00	1,259,058	393863	C_18_H_36_O_2_ (284)	2167	Antimicrobial activity [59]
**9**	**Octacosane**	17.417	57.00	216,706	95749	C_28_H_58_ (394)	2804	Potent antioxidant and anti-inflammatory [60]
**10**	**Eicosane**	17.517	57.00	257,731	77585	C_20_H_42_ (282)	2009	Antifungal activity [55,56]
**11**	**Pentadecane, 8-hexyl-**	17.809	57.00	423,598	114954	C_21_H_44_ (296)	2045	Microbicide activities [54]
**12**	**Triacontane**	20.132	57.00	417,739	87195	C_34_H_70_ (478)	3401	Antibacterial and Antifungal [61]
**13**	**1,2-Benzenedicarboxylic acid, mono(2-ethylhexyl) ester**	23.290	149.00	901,111	192559	C_16_H_22_O_4_ (278)	2162	antifungal compound [56,62]
**14**	**9-Octadecenoic acid (Z)-, 2,3-dihydroxy propyl ester**	27.527	55.00	954,576	81922	C_21_H_40_O_4_ (356)	2689	Antioxidant [63,64] anti-inflammatory insecticides [28,65]

**Table 3:** Antimicrobial activity of kefir grain powder 40 µg/mL, 50 µg/mL and 60 µg/mL against selected pathogens compared with 10 µg/mL ampicillin in terms of a zone of inhibition (ZOI).

Treatments	Conc.	*Salmonella* sp.	*E. coli*	*Streptococcus mutans*	*Candida albicans*	*Pseudomonas* sp.	*Klebsiella Pneumoniae*	*Staphylococcus aureus*	*Pseudomonas aeruginosa*
				ZOI (mm)					
**Kefir**	40 µg/mL	30.85 ± 0.21	23.7 ± 0.14	28.6 ± 0.2	15.7 ± 0.21	32.65 ± 0.27	36.8 ± 0.17	27.4 ± 0.33	18.6 ± 0.12
	50 µg/mL	32.25 ± 0.44	25.6 ± 0.19	32.3 ± 0.15	20.2 ± 0.11	35.35 ± 0.21	38.7 ± 0.22	31.2 ± 0.18	20.5 ± 0.22
	60 µg/mL	33.11 ± 0.24	33.4 ± 0.32	35.1 ± 0.24	23.25 ± 0.25	39.7 ± 0.28	43.25 ± 0.31	32.6 ± 0.32	32.6 ± 0.31
10 µg/mL Ampicillin (Control)	10 µg/mL	37.15 ± 0.28	27.5 ± 0. 5	28.9 ± 0.37	25.85 ± 0.25	32.85 ± 0.18	37.55 ± 0.18	36.1 ± 0.29	26.81 ± 0.3

All values were expressed as mean ± SEM (*n* = 3).

**Table 4:** Antifungal potential of kefir extract (100 mg/mL) against selected pathogenic fungal strains.

Fungal Strains	Growth Inhibition %
*Aspergillus flavus* AUMC 13922	80 ± 0.21
*Aspergillus fumigatus*	80 ± 0.7
*Aspergillus brasiliensis* AUMC 13921	75 ± 0.28
*Penicillium expansum*	87 ± 0.50

All values were expressed as mean ± SEM (*n* = 3).

## Data Availability

This investigation offers all the data collected or estimated throughout this.

## Sample Availability

Sample of the compounds are not available from the authors.

## Abbreviations

ALT	Alanine aminotransferase
AST	Aspartate aminotransferase
CREC	Carbapenem-resistant Enterobacter cloacae
HCV	Hepatitis C Virus
HBV	Hepatitis B Virus
HBsAg	HBV surface antigen
HBeAg	HBV core antigen
HBx	HBV pleiotropic regulatory protein X
GES-1:	Gastric epithelial cells
IL-10	Interleukin-10
LPS	Lipopolysaccharides
TNF-α	Tumor necrosis factor
INF-γ	Interferon-gamma
PBMCs	Peripheral blood mononuclear cells
FBS	Fetal bovine serum
qPCR	quantitative polymerase chain reaction
V.A	Vanillic acid
ZOI	Zone of Inhibition
